# Experiences and needs of home caregivers for enteral nutrition: A systematic review of qualitative research

**DOI:** 10.1002/nop2.990

**Published:** 2021-07-17

**Authors:** Jingjing Mou, Jianan Sun, Rui Zhang, Yang Yang, Wenwen Yang, Xiaosu Zhao

**Affiliations:** ^1^ The First Hospital of Jilin University Changchun Jilin Province China

**Keywords:** caregivers, enteral nutrition, nursing, relationship change, systematic review

## Abstract

**Aims:**

To systematically identify, evaluate and synthesize the qualitative evidence on enteral nutrition of home caregivers.

**Design:**

A qualitative evidence synthesis using the Sandelowski and Barroso methodology.

**Data Sources:**

We reviewed articles from eight databases: CINAHL, Embase, PubMed, Web of Science, Cochrane, CNKI, Wanfang Data and CSTJ. Qualitative, peer‐reviewed, original studies published in English or Chinese before April 2020 on home caregivers’ experience and needs for enteral nutrition were included. The studies were selected by screening titles, abstracts and full texts, and the quality of each study was assessed by two researchers independently.

**Review methods:**

Two researchers independently used qualitative assessment and review tools for quality assessment and thematic synthesis for data analysis.

**Results:**

This review included 10 articles. The themes identified included balance the enteral nutrition, the experiences and feelings in practice and the recommendations to meet challenge.

**Conclusion:**

Home caregivers reported that they played an important role and faced greater pressure. Future studies should establish a systematic and standardized follow‐up schedule to improve home caregivers’ physical and mental health.

**Impact:**

The findings established that home caregivers experienced not only changes in their roles and concerns but also spiritual changes. Home caregivers develop different coping strategies to adapt to enteral nutrition without standardized training and support. Although home caregivers make much account of enteral nutrition and feeding issues, they lack of information and support services. Understanding existing problems from a caregiver's perspective can allow interventions to be more clearly developed and well‐established training standards established in the future.

## 1 INTRODUCTION

In recent years, there has been a statistically significant increase in the number of people worldwide suffering from dysphagia, including individuals with neurological disorders and various cancers, who cannot eat orally or cannot meet their nutritional needs while recovering (Green et al., 2019; Martin and Gardner, 2017). Enteral nutrition, as an alternative to oral nutrition support, can not only provide sufficient energy for the body (Johnson et al., 2019; Welbank and Kurien, 2020) but also carries a lower risk of infection than parenteral nutrition, improve the immunity of the body and improves the prognosis of patients. Therefore, enteral nutrition has been widely used and supported (Bicakli et al., 2019; Zapka et al., 2014). At present, enteral nutrition is achieved by extending a tube into the stomach or small intestine. Nasogastric tube or nasoenteric tube is suitable for short‐term enteral nutrition. Nasogastric tube extends from the nostril to the stomach, and nasoenteric tube extends from the nostril to the jejunum of the small intestine. Gastrostomy or jejunostomy is used for long‐term enteral nutrition in which a tube directly passes through the abdominal wall into the stomach or small intestine (Baiu and Spain, 2019; Druml et al., 2016). Enteral nutrition is not only changing the lifestyle of patients but also has a huge impact on the family (Gea Cabrera et al., 2019; Pars and Soyer, 2020). Therefore, Enteral nutrition is an abnormal stimulus to home caregivers that may affect their cognitive, lifestyle and mental health (Villar‐Taibo et al., 2017). Specifically, home caregivers who lack skills and professional training are more overwhelmed with care (Jukic et al., 2017).

### 1.1 Background

Adequate nutrition support has a profound impact on patient recovery and safety, and is widely believed to save substantial cost (Chapman et al., 2015). With the development of medical teams and the improvement of community health care, after the condition of most patients receiving enteral nutrition becomes stable, the caregivers will choose to shift from hospital‐centred care to family‐centred care (Gramlich et al., 2018; Maeda et al., 2019). Enteral nutrition at home can not only reduce the waste of medical resources, infection complications and medical expenses but also can help to restore the independence of patients and families (Klek et al., 2014; Wong et al., 2018). In addition, medical institutions believe that enteral nutrition at home can reduce the bed load of large hospitals and the consumption of medical resources to make full use of the health resources of primary medical institutions in the community and promote the reasonable development of medical service institutions (Alivizatos et al., 2012). Caregivers believe that follow‐up care at home can not only allow patients to return to their former life habits but also facilitate feeling cared for by family members and enhance a patient's confidence in recovery (Gramlich et al., 2018). In addition, home caregivers play an important role in enteral nutrition from the onset of illness to care at home, taking on the daily duty of enteral nutrition, including feeding, ostomy and piping care, the management of related complications, and the provision of emotional support (Mori et al., 2019; Penner et al., 2012).

A number of studies have shown that caregivers are prone to experiencing symptoms of burden due to the performance of their care duties, such as feeling tired, feeling unwell and having physical or psychological problems such as anxiety and depression (Green et al., 2019; Jukic et al., 2017; Lim et al., 2018; Nordin et al., 2015; Villar‐Taibo et al., 2017). Taking care of patients with enteral nutrition is even more of a challenge for caregivers who have to perform complex medical tasks. Therefore, paying attention to their experiences and needs not only helps to maintain the medical needs of the patients but also contributes to the physical and mental health of the caregivers (Gillick, 2013).

To our knowledge, there is no systematic review of qualitative studies on the experience and needs of home caregivers for enteral nutrition. By exploring the experiences and needs from caregivers’ perspective, we can listen to their voices and better understand the challenges they face.

## 2 THE REVIEW

### 2.1 Aims

The review of qualitative studies aimed to identify and synthesize the available evidence for enteral nutrition in home caregivers. The questions guiding this review were as follows:(a)" what are home caregivers' perceptions and choices of enteral nutrition?", (b)" what are home caregivers’ daily care and priorities?" and (c)" what are home caregivers’ feelings and needs?"

### 2.2 Design

This systematic review of qualitative studies was conducted according to guidelines from the Center for Reviews and Dissemination (Tacconelli, 2010) and the Joanna Briggs Institute user guide (JBI, 2014). The qualitative studies were meta‐summarized and meta‐synthesized using the steps outlined by Sandelowski and Barroso (Sandelowski and Barroso, 2006).

### 2.3 Search methods

Electronic searches were conducted in April 2020 to identify studies published in English from CINAHL, Embase, PubMed, Web of Science, Cochrane, CNKI, Wanfang Data and CSTJ. The retrieval strategy involved Mesh terms and keywords related for "enteral nutrition" and "caregivers" in various databases. The search terms are listed in Supplementary File S1.

All the included studies in this review were consistent with the PICOS framework (Tacconelli, 2010): P =Patients, I = Phenomenon of interest, Co =context and S =types of studies (Table 1).

**TABLE 1:** Inclusion and exclusion criteria of the study selection

Inclusion criteria	Exclusion criteria
Participants: home caregivers	Participants: healthcare providers or patients
Phenomenon of interest: experiences and needs of caregivers on the enteral nutrition	Phenomenon of interest: study does not address experiences and needs of caregivers about enteral nutrition
Context: all caregivers’ contexts	Context: caregivers’ results have not been described separately from the other participants’ results
Type of study: a qualitative study	Type of study: a quantitative study
A peer‐reviewed, original study published in English and Chinese	A study that is not original research; a study published in languages not including English and Chinese

### 2.4 Search outcomes

**FIGURE 1:**
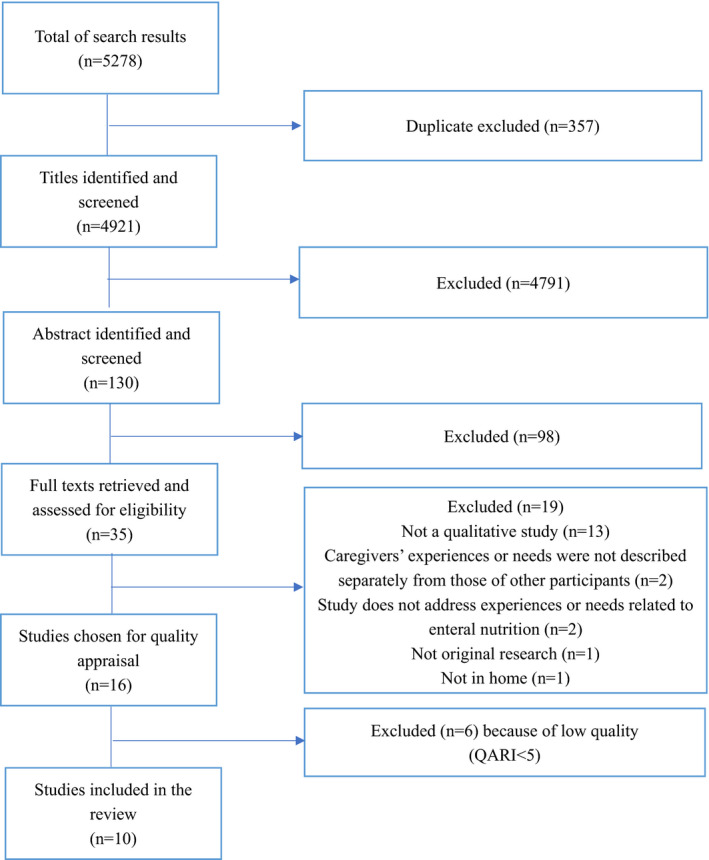
Study selection process of the original studies

A total of 5,278 studies were identified from electronic databases. To process the search results, the reference management programme EndNote was used (Bramer and Bain, 2017). First, duplicates (*N* = 357) were deleted (Tacconelli, 2010). A total of 4,921 studies were included a three‐step study‐selection process. In the study selection phase, the two researchers screened the titles (*N* = 4,921), the abstracts (*N* = 130) and the full texts (*N* = 35). Last, 10 studies were included. Table 2 provides information about the included studies. Disagreements between the researchers about a study's eligibility were resolved by a third researcher. The selection process is shown in Figure 1.

**TABLE 2:** Extracted data from the studies selected for the review

Authors and country	Purpose	Participants	Methodology: data collection and analysis	Key findings	Quality assessment
Green et al. (2019) UK	To understand the experience of caregivers in caring for patients with enteral tube and their views of supporting services and enteral tube‐related hospital admissions.	19 patients with enteral tubes;15 carers	Semi‐structured, face‐to‐face interviews; qualitative inductive descriptive design	Most hospital admissions can be avoided; Nurses were becoming more skilled at managing enteral tube. Enteral tube brought the hope of survival. Medical resources were abundant.	7
Mooi and Ncama (2020) South Africa	To explore the caregiver's needs about home‐based enteral nutritional therapy	3 patients; 4 caregivers	Semi‐structured individual interviews; content analysis approach	Caregivers need socioeconomic and psychosocial support to meet their needs.	8
Green et al. (2019) UK	To explore experiences of caregivers living with the tube and manage problems	19 patients; 15 caregivers	semi‐structured in‐depth interviews; thematic analysis	Participants need to adapt to a new lifestyle. Participants reported spending much time and effort to managing tube problems without support from healthcare practitioners.	7
Ang et al. (2019) Singapore	To explore perceptions of different modalities of long‐term enteral nutrition.	9 patients; 9 carers	semi‐structured interviews; content analysis	Some factors, such as previous experience, body image changes, and quality of life, influence the choice of enteral tube. Caregivers need knowledge, emotion, and follow‐up support from healthcare providers and from the community.	9
Asiedu et al. (2018) USA	To explore experiences of administering feeds during HEN	10 patients; 8 caregivers	photo‐elicitation interview; layered analysis	The caregiver developed a "cookie sheet system" for daily care. Caregivers were intimately involved in the patient's tube feeding.	7
Gil et al. (2018) Israel	To probe the considerations underlying the decision for gastrostomy	17 caregivers	in‐depth interviews; thematic approach	Enteral nutrition can save lives. Religious reasons influence the choice of enteral nutrition.	7
Alsaeed et al. (2018) UK	To explore the caregiver's experience with medicine administration	42 carers	online survey; thematic analysis	Improper drug formulation is likely to cause tube blockage. Caregivers develop resilient strategies for managing medications.	7
Jukic et al. (2017) Italy	To describe the views, experiences and adaptations of caregivers to enteral nutrition	30 caregivers	Hybrid research methods Qualitative research methods: focus group; thematic analysis	The carers gradually adapted to this life. Caregivers seek skills and are eager for healthcare support.	9
Penner et al. (2012) Canada	To explore the caregiver's life experience	6patients;6 caregivers	Two in‐depth interviews; Spiegelberg's three‐step approach	Caregivers take on new roles to care patients. Caregivers make lifestyle changes such as dropping social contacts and changing jobs.	8
Bjuresäter et al. (2012) Sweden	To explore what it means to be a close and how they can manage enteral tube	12caregivers	open interviews;	Families lose unity and joy at meals and socializing. Lovers can not pursue physical intimacy.	8

### 2.5 Quality appraisal

To determine the quality of each study and to increase the reliability of the assessment, the two researchers conducted a critical assessment based on the full texts of the selected studies (*N* = 35) (Tacconelli, 2010). To assess the quality of these studies, we used the Joanna Briggs Institute (JBI) Qualitative Assessment and Review Instrument (QARI), which comprises ten criteria. The original study must meet five of the criteria to be included in this systematic review. Quality was quantified by awarding 0 point or 1 point for each criteria. One of the studies was a mixed study, and only the qualitative part was included. On the basis of the quality evaluation, 10 studies were ultimately included in this review. Supplementary File S2 provides a quality assessment of the included studies.

### 2.6 Data extraction

The processing of research materials began with the extraction of data. Based on the PRISMA checklist, first the extracted data included study characteristics (author, year, country, purpose, participants, methodology,) and findings (primary themes, subthemes, or key findings) (Moher et al., 2009). Some of the included studies included the views of others, so only the caregivers' views were chosen to be explicitly expressed in the extraction. In addition, one of the studies, a mixed study, only extracted the caregivers' perspective from the qualitative study (Alsaeed et al., 2018).

### 2.7 Synthesis

The data analysis method adopted was thematic analysis, which is a systematic and objective method to analyse different types of literature or collected data. Data synthesis was conducted using the Sandelowski and Barroso's two‐step approach to synthesise (Sandelowski & Barroso, 2006). Meta‐summaries were conducted through the extraction, separation, grouping and abstraction of text findings into numbers and statement sets. The findings were extracted from each included study, and the details of these contributions to this review are listed in Table 3. Meta‐syntheses were performed by two reviewers independently to categorize and thematize home caregivers’ attitudes, experiences, feelings, recommendations and needs about enteral nutrition into main themes. First, all qualitative data were extracted from the papers and grouped by identifying questions with similar concepts. Second, similar concepts were grouped into preliminary descriptive themes. The syntheses were carried out by two researcher independently. Any disagreement that arose were discussed between the two reviewers until an agreement was reached.

**TABLE 3:** Inter‐study contribution to themes

Study	Themes						
	Balance the enteral nutrition		The experiences and feelings in practice			Recommendations and needs to meet challenge	
	Multiple factors to weigh	Select the most suitable one	Daily care	Lifestyle change	Relationship change	Healthcare system support	Social support
Green et al. (2019)			**×**			**×**	**×**
Mooi and Ncama (2020)						**×**	**×**
Green et al. (2019)	**×**		**×**			**×**	
Ang et al. (2019)	**×**	**×**	**×**			**×**	**×**
Asiedu et al. (2018)		**×**	**×**	**×**	**×**		
Gil et al. (2018)	**×**						
Alsaeed et al. (2018)	**×**		**×**				
Jukic et al. (2017)				**×**	**×**	**×**	**×**
Penner et al. (2012)				**×**			
Bjuresäter et al. (2012)			**×**	**×**	**×**	**×**	

X=Extracted qualitative data reflects theme.

## 3 FINDINGS

### 3.1 Description of the included studies

**FIGURE 2:**
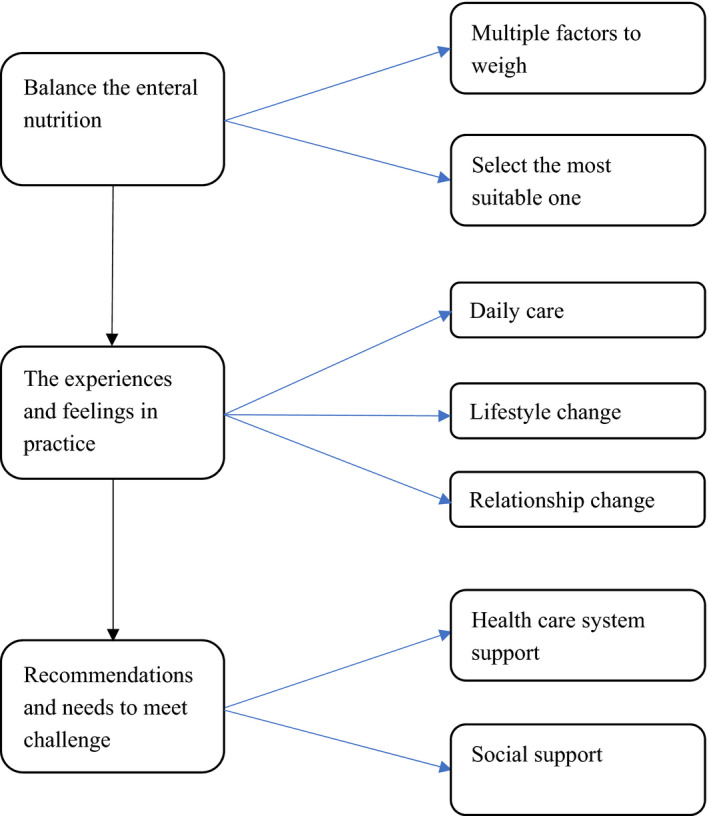
Themes and subthemes identified

Ten studies were selected for a systematic review, including three from the United Kingdom and one from the United States, Singapore, South Africa, Israel, Italy, Canada and Swede. In these studies, the number of caregivers involved ranged from 4 to 30, a total of 158 caregivers (Alsaeed et al., 2018; Ang et al., 2019; Asiedu et al., 2018; Bjuresäter et al., 2012; Gil et al., 2018; Green et al., 2019; Green et al., 2019; Jukic et al., 2017; Mooi and Ncama, 2020; Penner et al., 2012). Qualitative research methods were used in all the studies, including structured or semi‐structured interviews. All participants were home caregivers, most of them were family members of patients, and a few were employed caregivers (formal caregivers). These patients mainly needed enteral nutrition due to stroke (Mooi and Ncama, 2020), dementia, gastrointestinal tumour (Ang et al., 2019), neuromuscular disease (Mooi and Ncama, 2020) leading to the inability to eat or swallow normally, and malnutrition. The data of interest were evaluated and synthesized into three themes and seven subthemes (Figure 2). These themes of experiences and needs were outlined through the choice and use of enteral nutrition as influenced by its inherent complexity. Key findings and participants’ quotes were as follows.

### 3.2 Balance the enteral nutrition

Home caregivers reported that how to balance the enteral nutrition when they talk about patients’ physical condition, which included factors influencing caregiver choice of enteral nutrition, caregiver's selection of enteral nutrition types.

#### 3.2.1 Multiple factors to weigh

When caregivers made decisions on the application of enteral nutrition management, the recommendations of the medical team, the wishes of the patient, religious beliefs, etc., would influence the decisions of caregivers on enteral nutrition. As healthcare providers teach about the ways and necessity of enteral nutrition, most caregivers embrace the medical team's advice with the hope of providing patients with adequate energy (Ang et al., 2019; Asiedu et al., 2018; Gil et al., 2018). However, some family members also said they would seek advice from patients. For example, some patients would try their best to take oral food or even eat more through the mouth to maintain their weight, with enteral nutrition being used as a last resort. As one participant put it, "The staff doesn't want him (the patient) to stutter anything… Eventually, a compromise was reached where he was allowed to eat only a thickened liquid to ensure his quality of life, and he ate as much as he could, eating five 125 ml servings of muddy food a day." (Green et al., 2019). In addition, caregivers who have religious beliefs saw enteral nutrition as a gift from God that kept their families alive (Gil et al., 2018).

#### 3.2.2 Select the most suitable one

The selection of different types of enteral nutrition tubes was not only related to the length of time needed for enteral nutrition but also affected by previous experience and appearance. For patients with previous nasogastric tube experience, most caregivers believed that nasogastric tubes affected the patient's quality of life and self‐care except for causing aspiration and bleeding. As one participant described the patient, "Sometimes he (the patient) got angry, the nasogastric tube was in the nose, and bathing was troublesome." In addition, most caregivers, from the patient's perspective, believed that after the installation of a nasogastric tube or nasoenteric tube, the patient would refuse to socialize due to the high visibility of the tube, thereby affecting the patient's quality of life. The caregivers themselves could better master the management skills of gastrostomy due to their previous experience of nasogastric tube feeding and even thought that gastrostomy was easier to manage. Therefore, most caregivers choose gastrostomy (Ang et al., 2019; Asiedu et al., 2018). However, some caregivers were concerned about the risk to the patient from the operation, so they chose nasogastric or nasoenteric tubes. As one caregiver put it, "The patient was 86, had diabetes, and there was a risk to gastrostomy after anaesthesia." (Ang et al., 2019).

### 3.3 The experiences and feelings in practice

The experiences and feelings of caregivers were divided into three categories: daily care, lifestyle change and family relationship change.

#### 3.3.1 Daily care

Caregivers took responsibility and gave unconditionally to the patient not only out of love and care but also out of expectations for the patient and others around them (Bjuresäter et al., 2012). Caregivers made enteral nutrition part of their daily care by making lists, systematically preparing ingredients, managing nutrient pumps, flushing pipes, administering medication on time and performing ostomy care. As one participant described it, "Every morning when I got up, I had to take the nutrition bag off him, took the pump off and prepared a tray with his medication and specific food. When he got up, I would measure his blood sugar, then I would get water through the pipe, I would get medicine, and finally I would give him certain foods that would help him take in calories and other things. I would flush the tube again after I finished eating." (Green et al., 2019). It started out messy, but over time, the caregiver could drop the list and move on (Asiedu et al., 2018; Green et al., 2019). In addition, caregivers accompanied patients to appointments, helped them get dressed and provide emotional support to help them accept and adapt to their new lives (Asiedu et al., 2018).

In terms of drug administration, even if the caregiver used a syringe to flush the pipe before and after drug administration and dissolved the drug in warm water as prescribed by the doctor, he/she would still encounter problems such as pushing hard and blocking the pipe due to the combination of multiple drugs. Generally, the caregiver used carbonated drinks to flush the pipe or asked for help from the medical worker according to the guidelines (Green et al., 2019). In addition, to avoid the occurrence of these problems, some caregivers developed corresponding elastic strategies by themselves, such as dissolving drugs with hot water and dissolving drugs in a syringe (Alsaeed et al., 2018).

Almost all caregivers had witnessed enteral nutrition leading to physical discomfort, pain, ostomy infection, bleeding, duct release, granulation tissue growth, aspiration and other complications in patients (Ang et al., 2019; Green et al., 2019). At the beginning, caregivers were afraid of these problems and actively sought help from communities and hospitals. Over time, caregivers decided that most problems could be prevented or handled on their own without having to go to the hospital (Green et al., 2019; Green et al., 2019).

#### 3.3.2 Lifestyle change

Enteral nutrition made caregivers to take on new roles. Enteral nutrition was often the centre of daily life and work (Penner et al., 2012), which not only made caregivers physically exhausted but also affected their quality of life, resulting in a statistically significant reduction in leisure time companying their spouse and children (Jukic et al., 2017). In addition, physical exhaustion and emotional disturbances led to sleep disturbances in caregivers (Penner et al., 2012). To ease the burden, some families assumed the role of caregiver by more than one person, one of whom distributed and coordinated the role of other caregivers (Jukic et al., 2017). However, in addition to trying hard to adapt to the new living environment, employees (formal caregivers) also needed to gain enough knowledge and skills to take care of patients (Jukic et al., 2017). Although caregivers received enteral nutrition for varying periods of time, they eventually incorporated the task of enteral nutrition into their daily lives (Asiedu et al., 2018).

Taking care of patients requires considerable time and experience, and some caregivers might change or quit their jobs (Penner et al., 2012). In addition, to avoid the embarrassment and discomfort caused by not being able to eat by mouth when the patient attends a banquet, the caregivers would also avoid social situations and accompany the patient in as many aspects as possible (Bjuresäter et al., 2012; Penner et al., 2012).

#### 3.3.3 Relationship change

Couples avoided physical contact because of the location of the pipes and the smell of their mouths. In addition, because the patient cannot eat with the family, families lost their sense of belonging and happiness (Bjuresäter et al., 2012). Although enteral nutrition brought heavy burden and harm to the family, there were also unexpected gains, especially between children and patients, who snuggled up to the patient (father, uncle) and fed them, creating sweet moments and increasing intimacy (Asiedu et al., 2018). In addition, most caregivers did not want to put patients in nursing homes, and took on the role of caregiver, which also helped to rebuild and warm up the family. As one caregiver said, "Now our roles were reversed and I was happy to look after her (mother)." Taking care of patients was not only a moral and obligatory affirmation but also made the family feel the warmth of each other (Jukic et al., 2017).

### 3.4 Recommendations and needs to meet challenge

Based on their enteral nutrition suggestions, the recommendations were divided into two categories: healthcare system support and social support.

#### 3.4.1 Healthcare system support

Despite the education by healthcare professionals on enteral nutrition‐related issues and involvement throughout the treatment process, many caregivers still felt abandoned by the healthcare system, especially after patients left the hospital (Bjuresäter et al., 2012; Green et al., 2019; Green et al., 2019; Mooi and Ncama, 2020). Caregivers believed that improved healthcare system support could better meet caregivers' knowledge needs. In one study (Green et al., 2019), more than half of the respondents said they felt isolated and helpless at home, especially when complications arose, and did not know whom to turn to for help. They wanted specialized medical facilities and staff to help them when necessary. They also expected to communicate more with healthcare professionals to solve the confusion of how to manage enteral nutrition (Green et al., 2019). In addition, the follow‐up team was short of doctors and consisted of nutritionists and nurses. Home caregivers expected doctors to join them and provided relevant professional and guiding advice (Jukic et al., 2017). Finally, some caregivers were filled with fear when they started enteral nutrition care. However, medical staff often lack emotional education and focus on imparting knowledge, leading to mutual misunderstanding and disputes. Medical staff were expected to provide psychological comfort and emotional support (Ang et al., 2019; Green et al., 2019).

#### 3.4.2 Social support

Most families expressed a need for social support to help caregivers deal with related problems and ease their family burden (Jukic et al., 2017; Mooi and Ncama, 2020). In one study (Mooi and Ncama, 2020), most families could not afford enteral nutrition and needed financial support to survive. In addition, caregivers hoped to obtain get help and support from social institutions, thus giving to give patients the opportunity to study and work outside the home, and to establish a corresponding infrastructure to ensure the care of patients (Jukic et al., 2017). Caregivers also expressed a desire for help and support from relatives and friends, especially if the caregiver had an emergency to deal with (Jukic et al., 2017). At present, social volunteers and experienced caregivers often offer assistance to other patients receiving enteral nutrition who are willing to share their experiences and help others (Ang et al., 2019). However, although some developed countries had better healthcare systems, there might even be surplus and wasted resources, especially nutrients. Caregivers believed that governments were supposed to develop standardized, systematic delivery programmes (Green et al., 2019; Jukic et al., 2017).

## 4 DISCUSSION

The purpose of this study was to explore the experiences and needs of home caregivers for enteral nutrition. Three main themes were created to explain this phenomenon: Balance the enteral nutrition, the experiences and feelings in practice, and recommendations and needs to meet challenge.

The results show that when adequate nutrition cannot be obtained by oral administration, most caregivers choose enteral nutrition as an adjunct to nutrition. Enteral nutrition can not only provide enough energy but also help to maintain the normal physiological function of the body (Abunnaja et al., 2013). Some conscious patients weigh the importance of quality of life and longevity to choose whether to use enteral nutrition (Smith et al., 1999). When the patient is unconscious, delirious or has cognitive impairments, most guardians are inclined to provide active treatment and choose enteral nutrition (King et al., 2019). Therefore, it is necessary to explore the opinions of enteral nutrition from the perspective of the patients and caregivers.

In addition, in terms of the choice of enteral nutrition mode, caregivers went through tough choices (i.e. inadequacy, worry, guilt, unpreparedness and isolation). For patients who need enteral nutrition for a long time, caregivers realize that nasogastric or nasointestinal tubes are likely to lead to complications such as aspiration, catheter prolapse and nasal injury and limit self‐care and reduced social interaction of patients (Zaherah Mohamed Shah et al., 2012). In contrast, gastrostomy or jejunostomy has been reported to be well tolerated over the long term and can be as normal as possible. For these reasons, caregivers are better able to understand the benefits of ostomy (Wang et al., 2014). However, in the Asian context, the puncturing of skin via ostomy is considered unacceptable by caregivers (Ang et al., 2020; Yeh et al., 2010). Therefore, healthcare professionals should tailor discussion groups based on culture and state of an illness to help caregivers understand enteral nutrition.

In managing enteral nutrition, caregivers must not only complete their daily feeding but also be "smart" caregivers (Alsaeed et al., 2018). In the face of a series of enteral nutrition challenges, caregivers should establish a systematic and personalized process on the one hand to avoid feeding failure; on the other hand, careful observation and reflection are needed to avoid complications. Some caregivers have developed their resilience strategies aimed to ease of use. However, there may be potential problems with these strategies, so caregivers should communicate with healthcare professionals in a timely manner to ensure the safety of flexible strategies (Joos et al., 2015). Therefore, professionals strengthen regular training for caregivers and ensure adequate training knowledge.

To ensure the smooth operation of enteral nutrition at home, the healthcare system should improve the corresponding intervention measures. During a patient's hospitalization, healthcare workers should educate and train caregivers on enteral nutrition to improve their acceptance and competence (Jukic et al., 2017). After the patient was discharged from the hospital, a follow‐up mechanism was established. The follow‐up team should be composed of doctors, nutritionists, nurses, pharmacists and other disciplines and provide effective and personalized support and services according to the needs of patients and caregivers (Dinenage et al., 2015). In addition, follow‐up teams formed by communities and hospitals should communicate in a timely manner, establish unified operating standards and procedures, and avoid duplication of work or inconsistent information provision (Dinenage et al., 2015; Holst and Rasmussen, 2013). Moreover, multi‐team contributes to the development of discipline (Green et al., 2013).

In addition to provide operational training, psychological and financial support was also essential (Allen et al., 2014). The stress of caregivers at home correlates with caregiver behaviour, and the lower the stress, the higher the quality of care. However, due to lack of sleep and their ongoing responsibilities, caregivers might fail to perform well in their roles as employees, and being “good partners,” “filial children” and may be reprimanded by their employers or family members. Therefore, for some families, caregivers described themselves as trapped in the role of caregiver and are unable to maintain a normal life and communication, thus causing great physical and psychological harm to caregivers (Stajduhar et al., 2010). Healthcare workers should attach great importance to the psychology of caregivers. Multidisciplinary intervention, involving a psychologist can not only prepare the caregivers but also improve the effectiveness of enteral nutrition (Silver et al., 2004; Villar‐Taibo et al., 2017). In addition, mutual support and help between home caregivers of patients can not only provide caregivers with psychological comfort and reduce the burden but also allow them to learn from each other and share experiences (Dew et al., 2014). Furthermore, the study showed that social support for enteral nutrition was limited and health and financial resources were not met. Governments should increase coverage and efforts to reduce family financial burdens, optimize hospital‐community services and strengthen follow‐up teams to help resolve systemic barriers (Allen et al., 2014; Wong et al., 2018).

Future research should explore problem‐oriented approaches to guide healthcare professionals in designing specific interventions, providing caregivers with appropriate guidance and services, and helping caregivers to be more proactive in seeking help and working with healthcare professionals.

### 4.1 Limitations

This review has several limitations. First, with the inclusion of developed and developing countries, the health service system was quite different. Second, in the included studies, there were two studies from the United Kingdom in the same study, which might lead to duplication of the results. However, as this was a review of qualitative research, these two studies were included to comprehensively collect the experiences and needs of home caregivers on enteral nutrition. Third, excluding articles published in other languages might lead to missing some important information, but it could also avoid misunderstandings caused by inadequate understanding of other languages.

## 5 CONCLUSION

Many studies have focussed on providing support to patients. This systematic review reviews the experience and needs of home caregivers transitioning from exposure to enteral nutrition to competence in the home and emphasizes that home caregivers are not adequately supported. Barriers to training caregivers remain. These results suggest that we should strengthen the training of home caregivers, and establish unified and standardized training and services in hospitals and communities to reduce the burden of home caregivers.

## CONFLICT OF INTEREST

All authors can declare that there is no conflict of interest in this research paper and that this paper has not been published or is being considered for publication elsewhere.

## AUTHOR CONTRIBUTIONS

JJM and XSZ designed the study. JJM, YY and XSZ wrote the manuscript. JNS, RZ and WWY supervised and revised the study. JNS and RZ provided financial support.

## RESEARCH ETHICS COMMITTEE APPROVAL

The review does not require any research ethics committee approval.

## Data Availability

The data used and analysed in this study can be obtained from the corresponding author or original articles for reasonable requirement.
